# The making of the oral microbiome in Agta hunter–gatherers

**DOI:** 10.1017/ehs.2023.9

**Published:** 2023-05-22

**Authors:** Begoña Dobon, Federico Musciotto, Alex Mira, Michael Greenacre, Rodolph Schlaepfer, Gabriela Aguileta, Leonora H. Astete, Marilyn Ngales, Vito Latora, Federico Battiston, Lucio Vinicius, Andrea B. Migliano, Jaume Bertranpetit

**Affiliations:** 1 Department of Anthropology, University of Zurich, Switzerland; 2 Institut de Biologia Evolutiva (CSIC-Universitat Pompeu Fabra), Barcelona, Spain; 3 Dipartimento di Fisica e Chimica, Università di Palermo, Italy; 4 Department of Health and Genomics, Center for Advanced Research in Public Health, FISABIO Foundation, Valencia, Spain; 5 CIBER Center for Epidemiology and Public Health, Madrid, Spain; 6 Department of Economics and Business, Universitat Pompeu Fabra and Barcelona Graduate School of Economics, Barcelona, Spain; 7 Faculty of Biosciences, Fisheries and Economics, University of Tromsø, Norway; 8 Lyceum of the Philippines University, Intramuros, Manila, Philippines; 9 School of Mathematical Sciences, Queen Mary University of London, UK; 10 Dipartimento di Fisica ed Astronomia, Università di Catania and INFN, Catania, Italy; 11 Complexity Science Hub Vienna, Vienna, Austria; 12 Department of Network and Data Science, Central European University, Vienna 1100, Austria; 13 Department of Anthropology, University College London, UK

**Keywords:** hunter–gatherers, oral microbiome, Neolithic transition, pathogen transmission

## Abstract

Ecological and genetic factors have influenced the composition of the human microbiome during our evolutionary history. We analysed the oral microbiota of the Agta, a hunter–gatherer population where some members have adopted an agricultural diet. We show that age is the strongest factor modulating the microbiome, probably through immunosenescence since we identified an increase in the number of species classified as pathogens with age. We also characterised biological and cultural processes generating sexual dimorphism in the oral microbiome. A small subset of oral bacteria is influenced by the host genome, linking host collagen genes to bacterial biofilm formation. Our data also suggest that shifting from a fish/meat diet to a rice-rich diet transforms their microbiome, mirroring the Neolithic transition. All of these factors have implications in the epidemiology of oral diseases. Thus, the human oral microbiome is multifactorial and shaped by various ecological and social factors that modify the oral environment.

## Graphical abstract

**Figure:**
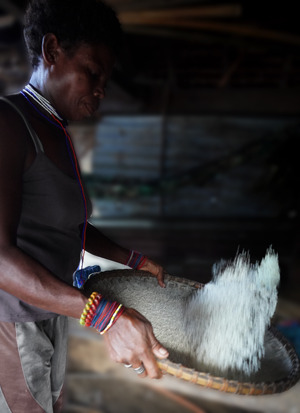

**Social media summary**: Our study shows how ecology, genetics and development shape the oral microbiome of Agta hunter–gatherers.

## Introduction

The composition and diversity of the human oral microbiota have been influenced by several factors during our evolutionary history (Cornejo Ulloa et al., 2019; Weyrich, 2021). Some are intrinsic biological characteristics of the host, such as age, sex and genetic composition, while others are external factors (Gomez et al., 2017; Willis et al., 2018), including diet, drinking water sources, oral hygiene, lifestyle and social interactions. Such factors modulate the physiological conditions of the oral cavity and affect the composition and diversity of oral microbiota. Many studies performed in farming and urban populations have indicated that while the oral microbiome is highly diverse between individuals, it remains stable over time (Costello et al., 2009). However, less is known about how the composition of the oral microbiome is modulated in hunter–gatherer populations, where the fully mature oral biofilm microbiome can be studied without the confounding effects of tooth brushing or professional dental cleaning (Velsko et al., 2019).

To investigate the multiple ecological and genetic factors shaping the human oral microbiome, we have analysed the oral microbiome of the Agta hunter–gatherers from the Philippines. The Agta were predominantly hunter–gatherers (fishing, hunting and gathering) when data collection took place (Page et al., 2018), and while their main source of animal protein was obtained by riverine and marine spearfishing or hunting, other activities such as inter-tidal foraging, wild food gathering, low-intensity cultivation, wage labour and trade complement their economy (Minter, 2010). There is high variability in the amount of hunting, gathering and sea foraging products traded for rice and other items (such as tobacco) with farming neighbours (Page et al., 2018). As a result, the Agta lifestyle ranges from completely mobile foragers with a protein-rich diet, to settled low-intensity farmers with a rice-rich diet (Page et al., 2016; Dyble et al., 2019).

To detect fine-scale variation in the oral microbiome of Agta hunter–gatherers, we collected saliva samples from 138 Agta aged 5–65 years (see Supplementary Table S1), sequenced the 16S rRNA region of oral bacteria and identified 5430 amplicon sequence variants (ASVs; see Callahan et al., 2017) belonging to 110 genera. Since 16S rRNA sequencing only provides relative abundance data, we refrained from making any inferences based on total yield. To study genetic host factors associated with microbiome composition, we also genotyped all individuals with the Axiom Genome-wide Human Origins array. We combined this information with additional individual data on household composition, age, sex and diet, measured as the proportion of meals including meat/fish (animal protein), and the proportion of meals that consisted exclusively of agricultural products (rice) (Supplementary Figure S1). Based on this rich dataset, we attempted to determine the contributions of age, sex, diet and host genetics in the making of the Agta oral microbiome.

## Results

### Factors influencing Agta oral microbiome composition

**Figure 1.**
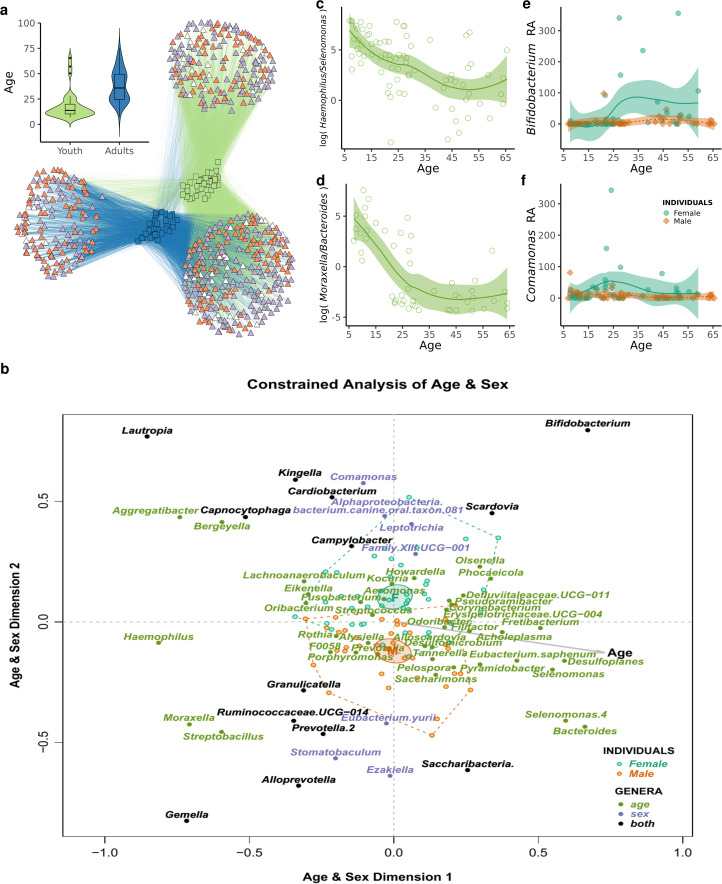
Age- and sex-related effects in the hunter–gatherer oral microbiome. (a) Network representation of the hunter–gatherer core measurable microbiota (CMM). Amplicon sequence variants (ASVs; triangles) are colour-coded as putatively pathogenic (purple), non-pathogenic (orange) or unclassified (white). Inset shows age distribution for the two clusters of individuals (squares). (b) Log ratio analysis constrained to age and sex differences on the bacterial composition at genus level. The effects of diet were partialled out. Only genera statistically significant in at least 20 (for age) or 10 (for sex) log ratios are displayed (*p*-value < 0.05 after Benjamini–Hochberg correction). Dashed lines enclose all individuals (dots) within a sex, with 95% confidence ellipses for their means. Taxa are colour-coded depending on the associated variable: age, sex or both. The starting point of the grey arrow indicates the mean age of the population (30 years old). Log ratio of (c) *Haemophilus* and *Selenomonas* relative abundance and (d) *Moraxella* and *Bacteroides* according to age. Line and shaded area indicate the 95% confidence interval of the mean. Relative abundance of (e) *Bifidobacterium* and (f) *Comamonas* according to age and sex. Lines and shaded areas indicate the 95% confidence interval of the mean for each sex.

The Agta oral microbiome is mostly composed of *Firmicutes* (mean relative abundance = 33.1%, SD = 11.4), *Proteobacteria* (27 ± 14.6%), *Actinobacteria* (15.5 ± 8.3%) and *Bacteroidetes* (14.5 ± 7.7%) (Supplementary Figure S2). To compare and identify the main ecological and social factors contributing to microbiome variation, we performed a constrained log ratio analysis (LRA; see Greenacre, 2018) on relative bacterial genus abundance. Age marginally explains 7.2% of the total log ratio variance (*p* < 0.0001, based on 9999 permutations), sex explains 2.2% (*p* = 0.018) and diet 3.6% (*p* = 0.015). Altogether they explain 13% of the total log ratio variance. We also applied a bipartite stochastic block model (biSBM) approach (Larremore et al., 2014) at the ASV level, where we assigned each bacterium to an individual, and then clustered individuals according to the bacteria they have in common. We restricted the analysis to the core measurable microbiota (CMM), that we define as ASVs present in at least 10% of the Agta to reduce random errors owing to low-prevalence taxa (Supplementary Figure S3). The best model produced two clusters of people and three clusters of bacteria (Figure 1a). While we did not find differences in diet or proportions of sexes between the clusters of individuals (Supplementary Figure S4), they strongly differ in their age distribution and define an adult cluster (mean age = 38 years old) and a young cluster (mean age = 18 years old) (Welch *t*-test, *t* = 5.78, d.f. = 71.35, *p* < 0.0001), with 55.48% of ASVs in the CMM being more associated with one of the clusters of individuals. The strong effect of age cannot be attributed to batch effects, as we found no association either between age and sampling time (Pearson *r* = −0.1452, *t* = −1.55, *p* = 0.1232), or between age and source (camp) of sampled water (Pearson *r* = 0.05, *t* = 0.58, *p* = 0.5618; see Methods for details of data collection). Thus, while age, diet and sex influence the composition of Agta microbiome, the biSBM singles out age as the main modulator of the hunter–gatherer core oral microbiome.

### Old age is associated with increased frequency of species classified as oral pathogens

To investigate the independent effects of ageing on the oral microbiome, we performed a LRA constrained to age and sex after partialling out the effects of diet. The resulting ordination shows that the effects of age and sex are mostly independent, with only a few genera being affected by both variables (Supplementary Figure S5). As expected, the first dimension is associated with age, while the second dimension splits individuals according to sex (Figure 1b).

There is a clear change in the composition and frequency of certain bacteria with age (Figure 1c and d). At a young age, we observe a higher relative abundance of organisms that typically live in mucosa, such as *Haemophilus* and *Moraxella*, which infect the upper and lower respiratory tract but are detected in the oral cavity and saliva as their vehicles of transmission. Other genera found at younger ages include bacteria normally associated with good oral health, such as *Bergeyella* and *Rothia* (Rosier et al., 2020). However, at older ages we observe a marked decline in the relative abundance of those genera and an increase in important pathogens related to periodontitis including the ‘red complex’ periodontal pathogen *Tannerella*, as well as other periodontitis-related bacteria (*Filifactor*, *Fretibacterium*, *Saccharimonas*, *Selenomonas* and *Phocaeicola*), consistent with a higher incidence of the condition with older age (Kassebaum et al., 2014). We also found organisms associated with cavities (*Olsenella*), dental plaque and dental calculus formation (*Corynebacterium*), pulmonary infections, sepsis or bacteremia, and chronic diseases (*Acholeplasma*) (see Methods for in-depth bacteria pathogenic classification). Another sign of ageing was the presence in the oral cavity of gut bacteria (*Bacteroides*), indicating a potential age-related decline in immunological function and filtering (De Maeyer & Chambers, 2021). However, such changes are not associated with a decrease in the alpha-diversity of the total oral microbiome as measured by the number of bacteria observed or their phylogenetic complexity (Supplementary Figure S6a and b), suggesting that the overall effect of ageing is a replacement of protective and commensal bacteria by putatively pathogenic ones. This is supported by an increase in the number of potential pathogenic bacteria in the CCM in bacterial clusters associated with older ages (Fisher exact test, *p* < 0.001) (Figure 1a).

### Sex differences shape composition but not diversity of the Agta oral microbiome

We found no differences in alpha-diversity in the Agta oral microbiome between males and females (Supplementary Figure S6c and d). It is possible that the high levels of equality in dietary composition, social interactions and mobility between men and women in the Agta society (Dyble et al., 2016; Migliano et al., 2017) may be associated with similarities in microbiome diversity between sexes. Nevertheless, the LRA constrained to age and sex shows sex-related differences in the composition of the oral microbiome (Figure 1b). For example, *Stomatobaculum* and *Eubacterium yurii* present in the oral cavity of smokers (Duan et al., 2017) are associated with males, consistent with Agta men chewing tobacco more frequently than women. It should be noticed that betelnut chewing is a widespread habit among the Agta, a practice linked in other populations to oral carcinogenesis (Oslam et al., 2019) and changes in the oral microbiome (Hernandez et al., 2017). Future studies should investigate potential links between betelnut chewing and oral microbiomes in Agta. It is also interesting to mention *Comamonas* (Figure 1f). Even if it has been reported as a possible contaminant in microbiome studies (Einsenhofer et al., 2019), its higher relative abundance in females could be related to the fact that it can degrade progesterone (Liu et al., 2013). This bacterium has been found in subgingival samples, where female hormones could be present either in saliva or in the gingival crevicular fluid.

### Age and sex interactions in microbiome composition

Some bacteria are significantly associated with both age- and sex-related differences, such as *Gemella*, which is a prevalent inhabitant of the respiratory mucosa besides *Haemophilus* and *Moraxella*, supporting the idea that mucosa-associated or respiratory-tract organisms are more frequently acquired in young individuals, especially males. At older ages, the *Bifidobacterium*/*Saccharibacteria* ratio differentiates by sex: while *Bifidobacterium* is associated with females, the periodontal pathogen *Saccharibacteria* is associated with males. Thus, the observed trend of an increase in periodontal pathogens with age is stronger in males, in line with the global epidemiology of the disease (Shiau & Reynolds, 2010; Eke et al., 2015). On the other hand, we found an increase in the relative abundance of caries-related pathogens *Scardovia* and *Bifidobacterium* associated with reproductive age females. Caries incidence increases with age and is more prevalent in females (Ferraro & Vieira, 2010), with a more saccharolytic or acidic salivary environment in older women, together with hormonal fluctuations and lower salivary flow (Zaura et al., 2017), possibly facilitating their proliferation. The strong association of *Bifidobacterium* with adult females could also be explained by its presence in breast milk (Fernández et al., 2020). Its proliferation (Figure 1d) also coincides with the start of the reproductive age and increase in childcare (Dyble et al., 2019).

### Effect of rice consumption

**Figure 2.**
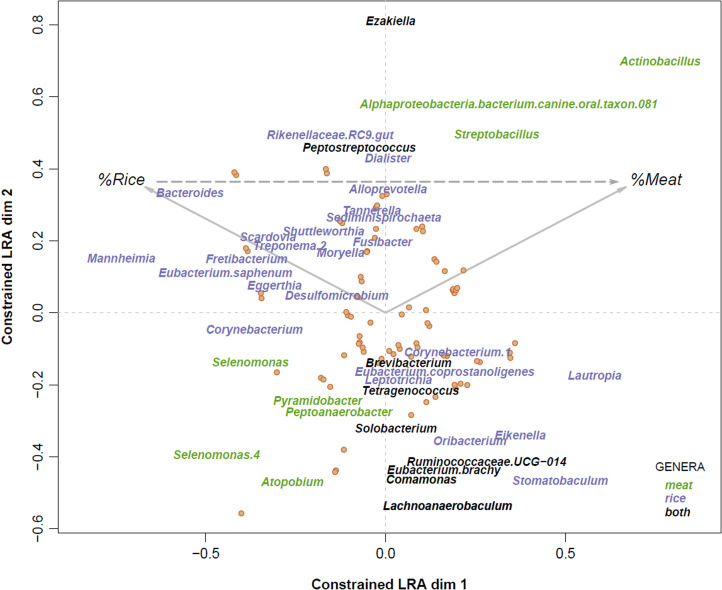
Effect of diet on the oral microbiome in the Agta. Log ratio analysis constrained to diet differences on bacterial composition at genus level. The effects of age and sex were partialled out. Only genera statistically significant in more than five (for rice) or three (for meat) log ratios are displayed (*p*-value < 0.05 after Benjamini–Hochberg correction). Taxa are colour-coded based on the variable they are associated with proportion of meals with meat (%Meat), proportion of meals with only rice (%Rice) or both. The original plot was slightly rotated without any change in explained variance, so that the dashed vector indicating the difference between %Meat and %Rice was horizontal.

While the impact of diet on gut microbiomes has been established (Turnbaugh et al., 2009; David et al., 2014; Schnorr et al., 2014; Smits et al., 2017), its role in the oral microbiome is still unclear. Some studies have found little or no effect, whereas others have found associations with specific nutrients (De Filippis et al., 2014; Belstrøm et al., 2014; Weyrich, 2021; Zaura et al., 2017). Variation in rice consumption in our sample allowed us to assess the effects of a hunter–gatherer diet and of the recent introduction of farming products on the Agta oral microbiome. We performed an LRA on relative bacterial genus abundance after partialing out the effects of age and gender (Figure 2). The first dimension of the ordination is related to the transition from a diet where all meals include meat to where most consist of only rice. Agta following a meat-rich diet showed a higher relative abundance of *Actinobacillus*, *Alphaproteobacteria* and *Streptobacillus* and lower abundance of *Selenomonas*, *Atopobium*, *Peptoanaerobacter* and *Pyramidobacter*. The higher relative abundance of *Actinobacillus* in individuals ingesting a protein-rich diet is particularly interesting, given the extraordinary proteolytic potential of *A. actinomycetencomitans*, a well-known oral pathogen with destructive effects in the gingival tissue and in aggressive forms of periodontitis (Fives-Taylor et al., 1999). At the other extreme, in individuals with a rice-rich diet, there is an increase in the abundance of the highly saccharolytic dental caries pathogen *Scardovia*, of *Treponema*, of gut organisms like *Butyrivibrio* and Erysipelotrichaceae, and of *Eggerthia*, a rare organism isolated from dental abscesses. We also ranked ASVs based on whether they are more present than expected in individuals with a high or low proportion of meals with only rice or with meat and fish. We found that the scores associating bacterial species with either rice or meat and fish are negatively correlated (Spearman's *ρ* = −0.47, *p* < 0.0001). This fits with a general separation of oral microorganisms into saccharolytic (caries-related, acidogenic and acidophilic) and proteolytic (gum-disease and halitosis related, alkalophilic and NH_4_ generators), as suggested in a metabolome-based study (Zaura et al., 2017). Our results suggest that more settled Agta, who consume more rice, experience a decline in oral health, mirroring a general pattern of health decline owing to a Neolithic-like diet and a more farming-derived lifestyle (Page et al., 2016; Adler et al., 2013; Sabbatani & Fiorino, 2016).

### Pathogenic oral bacteria are associated with host collagen genes

The interaction between host genetic makeup and microbiome composition differs across body sites (Kolde et al., 2018; Blekhman et al., 2015), and seems especially weak in the oral cavity (Shaw et al., 2017), making it difficult to assess the co-evolution of our genome and the oral microbiome. We therefore performed a genome-wide association study (GWAS) using a mixed model approach in a hunter–gatherer population without the confounding influence of antibiotics or brushing. We treated the relative abundance of each bacterium as an independent trait, adding age, sex and household as covariates and kinship as a random effect. Household membership was used as proxy for the strength of social interactions between individuals, as social interactions predict microbiome sharing; for an extended analysis of the effects of sociality on the oral microbiome of the Agta population, see the companion article by Musciotto et al. (2023) in this issue.

**Figure 3.**
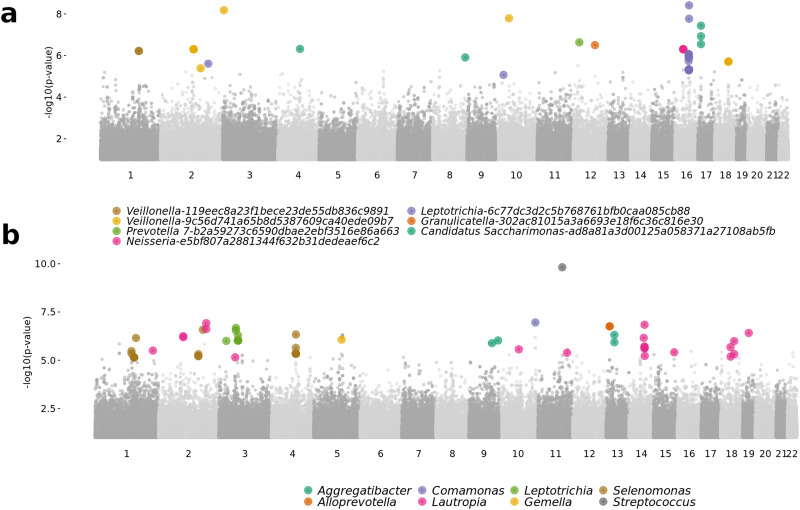
Genome-wide association study of bacterial abundance. Aggregated Manhattan plot of the GWAS results of (a) seven ASV and (b) eight genera with non-zero PVE (‘chip heritability’) estimates with at least one significant genetic association. Each dot is a single nucleotide polymorphism (SNP), and significant SNP–bacteria associations (*q* < 0.1) are colour-coded.

Analyses were performed using the CMM, and then using 92 genera present in at least 10 Agta. All bacteria identified in the Agta (Supplementary Table S2 and Supplementary Figure S7) overlap with those of other oral microbiome GWAS (Blekhman et al., 2015; Gomez et al., 2017; Kolde et al., 2018), pointing to a small subset of oral bacteria influenced by the human genome (Figure 3). A pathway enrichment analysis linked this subset to several biological host functions (body fat metabolism, wound healing and collagen trimmers; Supplementary Table S3). Of relevance is an association between the bacterial genera classified as potentially pathogenic (*Aggregatibacter* and *Selenomonas*) with genetic variation in collagen genes. The ability to bind collagen is a vital feature in the oral cavity, as many oral bacteria require collagen-binding proteins to attach to oral tissues (Mira et al., 2017) suggesting a genetic basis for the predisposition to bacterial biofilm formation. We further tested whether we could detect signatures of recent positive selection in the host genomic regions associated with the oral microbiome, but found no signal.

## Discussion

The Agta oral microbiome is influenced by external factors such as social interactions (Musciotto et al., 2023), as well as intrinsic and ecological factors including age, sex, diet and host genetics. Among the latter we identified age as the factor with the strongest effect on microbiome composition, with commensal or beneficial microbiota being replaced by potentially pathogenic ones with ageing. The proliferation of oral bacteria likely to be pathogenic exhibits sexual dimorphism, with caries-related (in females) and periodontitis-related (in males) bacteria increasing with age, possibly associated with immunosenescence (Preshaw et al., 2017) and a sex-specific oral environment resulting from biological and cultural factors. The increase in farming-derived novel foods such as rice also seems to have an effect on microbiome composition and host health. Overall, the relatively small subset of bacteria linked to the host genome suggests that the Agta oral microbiome is mainly affected by environmental (diet) and intrinsic factors (age), with little influence of individual host genetic variation (Mukherjee et al., 2021; see our Supplementary Figure S5). We conclude that the Agta Palanan oral microbiome is multifactorial with distinct subsets of bacteria shaped by specific ecological and social factors, reflecting multiple adaptations in the domains of life history, sociality and diet.

## Methods

### Ethics approval

This study was approved by the UCL Ethics Committee (UCL ethics code 3086/003) and carried out with permission from local government in the Philippines and Agta community members in Palanan. Informed consent was obtained from all participants, after group and individual explanation of research objectives in the indigenous language. A small compensation (usually a thermal bottle or cooking utensils) was given to each participant. The National Commission for Indigenous Peoples (NCIP), advised us that the process of Free Prior Informed Consent had to be obtained from community leaders, youth and elders under the supervision and validation of the NCIP. This was done in 2017 with the presence of all community leaders, elders and youth representatives at the NCIP regional office, with the mediation of the regional officer and the NCIP Attorney. The validation process was approved unanimously by the community leaders and the NCIP, and validated the full five years of prior data collection.

### Saliva sample collection

Saliva samples from 155 Palanan Agta were collected over two field seasons: April–June 2013 and February–October 2014 during the dry season. For comparative genetic studies we also used saliva samples from 21 Mbendjele BaYaka, an African hunter–gatherer population, collected in 2014, and 14 Palanan farmers collected in 2007–2009. In all cases saliva was collected using the Oragene⋅DNA/saliva kit and participants were asked to rinse their mouth with water and spit into a vial until half full. After collection and transportation, saliva samples were stored at the UCL Department of Anthropology, London, UK at −20°C.

### Microbial DNA extraction and 16S rRNA gene sequencing

DNA was extracted from 155 Agta saliva samples following the protocol for manual purification of DNA for Oragene⋅DNA/saliva sample. The 16S rRNA gene V3-V4 region was amplified by PCR with primers containing Illumina adapter overhang nucleotide sequences following the 16S Metagenomic Sequencing Library Preparation protocol for the Illumina MiSeq System. All PCR products were validated through an agarose gel and purified with magnetic beads. Index PCR was then performed to create the final library also validated through an agarose gel. All samples were pooled together at equimolar proportions and the final pool was qPCR quantified prior to the MiSeq loading. Raw Illumina paired-end sequence data were demultiplexed, quality filtered and denoised with QIIME 2 2019.1 (Bolyen et al., 2019) and DADA2 (Callahan et al., 2016). DADA2 generates single nucleotide exact amplicon sequence variants (ASVs). ASVs are biological meaningful entities as they identify a specific DNA sequence and allow for higher resolution than using operational taxonomic units (see Callahan et al., 2017). Taxonomic information was assigned to ASVs using a naive Bayes taxonomy classifier against SILVA database release 132 with a 99% identity sequence (Quast et al., 2013). ASVs that did not belong to the kingdom Bacteria were classified as mitochondrial or chloroplast sequences, and samples with an extremely low number of sequences (8000) were excluded from further analyses. ASVs were aligned with MAFFT (Katoh, 2002) and a rooted phylogenetic tree was constructed with FastTree2 (Price et al., 2010) using default settings via QIIME 2. This resulted in a total of 5430 ASVs and 138 Agta (67 women and 71 men). We generated a rarefaction curve with R package *vegan* version 2.5-7 (Oksanen et al., 2020) to determine that the richness of samples had been fully observed (Supplementary Figure S8). The number of observed ASVs and Shannon Diversity indexes were calculated with R package Phyloseq version 1.30.0 (McMurdie & Holmes, 2013). Faith's Phylogenetic Diversity index (Faith, 1992) was calculated with R package *picante* version 1.8.2 (Kembel et al., 2010) using the rooted phylogenetic tree generated in R (R Core Team, 2020). To determine the set of microbial traits to be included in the analyses, we selected ASVs with at least 10 reads in at least two individuals (*n* = 1980), then aggregated those with a taxonomic assignment at a genus level, resulting in 110 genera. At the ASV level we also defined a CMM, consisting of ASVs that appeared in at least 10% of the Agta individuals (14 or more) resulting in 575 ASVs (out of 1980) representing 90% of the composition of the Agta oral microbiome.

### Genotype data

A total of 190 saliva samples were genotyped with the Affymetrix Axiom Genome-Wide Human Origins 1 array. DNA extraction was carried out following the protocol for manual purification of DNA for Oragene⋅DNA/saliva samples in the same laboratory that sequenced the 16S rRNA data. Samples were analysed with Axiom Analysis Suite v4.0 following the Axiom genotyping best-practices workflow for saliva samples. 618,810 markers and 177 samples passed initial quality control. Single nucleotide polymorphisms (SNPs) with less than 95% genotyping rate and samples where the estimated gender from the genotypes did not match the recorded gender were excluded. Duplicated samples were identified with KING (Manichaikul et al., 2010) and removed. This resulted in a total of 617,063 markers and 174 samples: 141 Agta, 19 BaYaka and 14 Palanan farmers.

### Ethnographic data collection

Ethnographic data collection occurred over two field seasons from April to June 2013 and from February to October 2014. In the first season we censused 915 Agta individuals (54.7% male) across 20 camps, collecting basic information on household composition, sex and estimated ages. Following relative ageing protocols (Diekmann et al., 2017), accurate ages were established for all individuals after data collection.

### Diet data collection

Dietary recall data were collected at the household level over a period of 10 days. We asked the mother and father in the afternoon (between 17:00 and 18:00) which foods they had eaten on that day, including agricultural products traded with nearby farmers. We counted the total amount of meals recorded for a household and established what proportion of these consisted of meat, vegetables, fruits, honey and rice. Therefore, this is only a rough guide to dietary composition and does not take calorific intake or absolute weights of the different food types into account. Dietary data for 80 individuals (37 males and 43 females) were annotated based on the proportion of meals that consisted of only rice, and the proportion of meals that included meat (primarily fish and other marine resources and game).

### Classification of oral bacteria as pathogens

Bacteria were classified as potential oral pathogens if they have been reported as etiological agents of periodontitis or dental caries. Assignment as a periodontal pathogen was performed according to the systematic review of Pérez-Chaparro et al. (2014) and Socransky et al. (1998), or if they have been previously associated with gum disease (Camelo-Castillo et al. 2015; Khemwong et al., 2019). Bacteria were classified as caries pathogens if they were described in transcriptomic studies of human cavities, according to Sómon-Soro et al. (2014) and Sómon-Soro and Mira (2015), previously associated with caries (Tanner, 2015; Kressirer, 2017), cavities (Wolff et al., 2019) or dental plaque and dental calculus formation (Mark Welch et al., 2016; Ferrer & Mira, 2016). Bacteria reported as etiological agents of respiratory infections and biofilm-mediated infections were also considered pathogens, including organisms present in healthy carriers. These included species described in Leung et al. (2018), Bellussi et al. (2019) and Natsis and Cohen (2018). Bacteria causing urinary tract infection or sexually transmitted diseases transiently found in the oral cavity were also considered as potential pathogens and included microorganisms described in Lanao and Pearson-Shaver (2020) and Jung et al. (2017). Common oral commensals potentially causing endocarditis or systemic infections in immunocompromised patients were not considered pathogens. If a bacterium was isolated from the oral cavity of another animal species, it was considered oral for the sake of our classification. If taxonomic classification in our dataset could be assigned at the genus level only, it was considered potentially pathogenic if: (i) >90% of species within the genus were pathogenic; or (ii) it included a major pathogenic species but the other species within the genus were not oral according to the Human Oral Microbiome Database (http://www.ehomd.org/) (Chen et al., 2010; Escapa et al., 2018). Cases where taxonomic classification of the ASV was only possible at the family level or higher (order, class) or ASVs with a top hit to a sequence classified as ‘Oral taxa’ in databases but without a species assignment were not included in this classification (Supplementary Table S4). We cannot rule out the presence of relatively new genera not included in the database at the time of this study, such as *Schallia*.

The alternative classification of certain bacteria as ‘pathobionts’ rather than pathogenic has been proposed (Simón-Soro & Mira, 2015) to account for the fact that while some bacteria are associated with oral pathologies when present in high levels, they can also be found at low levels in healthy individuals. However, this does not prevent the identification of potential pathogens where there is strong evidence of the role of bacteria in creating the conditions for the development of oral disease. A clear example is provided by the role of the extremely saccharolytic, acidogenic and acidophilic *Streptococcus mutans* or *Scardovia wiggsiae* bacteria that proliferate in an acidic environment (either by causing it or being able to tolerate it), a known condition for the development of cavities. For this reason, we decided to rely on the categories of potentially pathogenic and non-pathogenic in our classification scheme.

### Multivariate compositional data analysis on microbial composition

We performed a constrained LRA using the R package *easyCODA* (Greenacre, 2018) on the Agta oral microbiome at the genus level using as constraining covariates age (continuous variable), sex (male and female) and diet (both proportion of meals with meat and proportion of meals with only rice). Microbiome abundance counts of each Agta individual were treated as compositional data (Gloor et al., 2017) and converted to logarithms of ratios (log ratios). Constrained LRA is a special case of redundancy analysis (Greenacre, 2018) where total log ratio variance is decomposed into fractions explained by covariates (the ‘constrained variance’) and a residual fraction. Then, ordination resulting from LRA explains a maximum of the constrained variance in a reduced two-dimensional solution. Statistical significance of the three covariates was assessed using a multivariate permutation test (999 permutations) in the R package *vegan*. There is no correlation between the three covariates, except within the diet covariate, where the two variables are negatively correlated (Spearman's *ρ* = −0.54, *p* < 0.0001; Supplementary Figure S1). To focus on the genera affected only by intrinsic factors (age and sex), we performed a constrained LRA on the microbial composition after partialling out the effects of diet. Similarly, to identify genera affected exclusively by diet, we performed a constrained LRA after partialling out the effects of age and sex. Taxon-covariate association was ranked by counting the number of significant log ratios for each of the taxa, with *p*-value < 0.05 controlling for false discovery rate (FDR) at level *α* = 0.05.

### Community detection

To model the relationship between the Agta and the CMM, we used a stochastic block model (SBM) approach specifically suited for bipartite networks (Larremore et al., 2014). SBM infers the community structure (Fortunato, 2010) that better fits the existing graph, by building a prior distribution for edges that holds no information on real data and using it in the framework of Bayesian inference (biSBM) to find a partition of the two types of nodes whose associated entropy is maximal. In this framework, the absence of links between nodes of the same type or set is not considered informative, as expected given the bipartite nature of the graph, different from the general version of SBM. We selected the number of clusters in the two sets that minimised description length (Peixoto, 2017). Robustness of the clustering was assessed by calculating the average adjusted Rand index (ARI) between iterations (*n* = 100), finding a mean ARI on Agta = 0.90 and a mean ARI on ASV = 0.70. The ARI measures the similarity of two partitions against a null hypothesis of random assignment maintaining the size of the different clusters; the closer to 1 it is, the more robust is the classification (Hubert & Arabie, 1985). The resulting clusters were plotted with *graph-tool* (Tiago, 2014).

### Ranking of bacteria associated with diet

ASVs were ranked from −1 to 1 based on whether they were more present than expected in individuals from a given category: low proportion vs. high proportion of meals with only rice, and low proportion vs. high proportion of meals with meat, based on the median value of the population for each variable. For example, a meat-associated score close to 1 indicates that an ASV is present above the expected in individuals with high proportion of meals with meat (above the median of the population). A rice-associated score near 1 indicates that an ASV is present above the expected in individuals with high proportion of meals that consist of only rice.

### Microbiome genome-wide association studies

We used a GWAS approach to identify specific SNPs associated with microbial abundance in the Agta using *GEMMA* version 0.94 (Zhou & Stephens, 2012). GWAS were performed using the relative abundance of a given taxon as a phenotype trait, adding as covariates age, sex and household (as a proxy for diet and shared environment). A kinship matrix calculated by KING using identical by descent segment inference (Manichaikul et al., 2010) was included as random effects. For the GWAS analyses, we applied the following quality control steps. First, to detect ancestry outliers, we filtered samples to keep only bi-allelic autosomal SNPs with minor allele frequency (MAF) > 5% and without missing data with *PLINK* 1.9 (Chang et al., 2015). This dataset was pruned for linkage disequilibrium using --indep-pairwise 50 5 0.2, and we performed a principal component analysis with *EIGENSOFT* version 7.2.1 (Patterson et al., 2006) to identify ancestry outliers and exclude them (Supplementary Figure S9). Second, per sample heterozygosity was calculated with *PLINK* and samples with overall increased or decreased heterozygosity rates (±3 SD from the mean of the population) were removed. A total of 129 Agta samples passed microbiome and genotype data quality controls and were included in the microbiome GWAS analyses. Analyses were done at genus level and on the CMM. The number of individuals tested ranged from 10 to 129 and the number of SNPs tested ranged from 270,569 to 313,198 markers depending on the taxon, as we included only samples with non-zero abundance, excluding SNPs with MAF < 10% and with more than 5% missing data. When we performed the GWAS at the genus level, we only included in the analysis the 92 genera present in at least 10 Agta individuals. *p*-Values were adjusted for multiple testing by FDR within each taxa (92 genera and 575 ASV), and SNP-taxa associations were considered significant at *q*-value < 0.1 when the proportion of variance in bacterial abundance explained by the genotypes (PVE or ‘chip heritability’) was non-zero. The proportion of variance in the phenotype (bacterial abundance) explained by the genotypes tested (PVE or ‘chip heritability’) was estimated for each taxon and was considered non-zero if the standard error measurements did not intersect zero. We applied genomic control to correct for cryptic relatedness and population stratification and minimise false positives induced by inflated association test statistics (Devlin & Roeder, 1999). To do so, we estimated the genomic inflation factor as the median value of the likelihood ratio test (LRT) values divided by 0.456 (median of a χ^2^(1) distribution) and recalculated the *p*-values after dividing LRT values by the genomic inflation factor (Bacanu et al., 2000). The threshold of significance was set at FDR 10%, and only genomic positions having at least three samples for the major homozygous genotype and for the heterozygous genotype were considered. SNPs were annotated with *ANNOVAR* (Wang et al., 2010) in *GRCh37* (hg19) using *RefSeqGene* and *dbSNP* 147. For the enrichment analyses, we extracted genes associated with all non-intergenic SNPs and classified genes in the background set (consisting of all genes in the Axiom Human origin array) and the set to test (all genes with a non-intergenic SNP significantly associated with an ASV or a genus). We performed a gene ontology enrichment analysis with *ViSEAGO* (Brionne et al., 2019) and *topGo* (Alexa and Rahnenfuhrer, 2019) R packages (Fisher exact test) and *FUMA GENE2FUNCTION* module (Functional Mapping and Annotation of Genome-Wide Association Studies; Watanabe et al., 2017) to perform pathway enrichment analysis (hypergeometric test) with FDR 5%.

### Selection analyses

To test whether GWAS SNPs showed any signal of recent positive selection we performed a genome-wide selection scan. We phased the Agta and Palanan farmer populations independently. For each population, samples identified as ancestry outliers by a principal component analysis or with overall increased or decreased heterozygosity rates (±3 SD from the population mean) were excluded. A total of 138 Agta samples were phased using *SHAPEIT2* version v2 (r900) (O’Connell et al., 2014) with the duoHMM method to improve phasing by integrating known pedigree information. SNPs with missing data were removed and window size was set to 5Mb. Due to the small sample size, to phase the 14 unrelated Palanan farmers we used SHAPEIT2 with default parameters and the 1,000 Genomes Phase 3 panel of haplotypes (Auton et al., 2015) as a reference dataset. SNPs with missing data were removed. For the selection analyses, we excluded one of each pair of related individuals by removing the individual with the lowest call rate in the Agta phased dataset. This resulted in 38 unrelated Agta individuals and 14 unrelated Palanan farmers. We ran the Integrated Haplotype Score (iHS) (Voight et al., 2006) on the Agta phased dataset, and the Cross-population Extended Haplotype Homozygosity (XP-EHH) test (Sabeti et al., 2007) comparing the Agta against Palanan farmers as implemented in *selscan* version v1.3.0 (Szpiech & Hernandez, 2014) to identify signals of positive selection in GWAS SNPs. Both tests were run with default parameters and with the genetic map provided by the 1,000 Genomes Phase 3 (Auton et al. 2015). To identify regions under selection, for each test we selected markers with scores in the 95th percentile that had at least three markers in the 99th percentile in the surrounding area (± 10 kb). For iHS we used absolute values, while only positive scores were analysed for XP-EHH.

## Research transparency and reproducibility

16S amplicon data (EGAS00001005317) are deposited at the European Genome-phenome Archive (EGA), which is hosted at the EBI and the CRG. Genome data generated in this study has been deposited at EGA under accession number EGAS00001005315. Data on age, household composition and diet that support the findings of this study are available on request from the corresponding authors (J.B. and A.B.M.). Individual data are not publicly available owing to them containing information that could compromise research participant privacy. Source code and data for visualisation are available at https://doi.org/10.5281/zenodo.6342212.

## Supplementary material

For supplementary material accompanying this paper visit https://doi.org/10.1017/ehs.2023.9. click here to view supplementary material
